# Bronchoscopic Diagnosis of Pulmonary Sarcoidosis Using Endobronchial Ultrasonography-Guided Transbronchial Needle Aspiration (EBUS-TBNA) and Transbronchial Lung Cryobiopsy (TBLC): A Safe and High-Yield Dual Approach

**DOI:** 10.7759/cureus.95356

**Published:** 2025-10-24

**Authors:** Hiroaki Ota, Hiroya Kawasaki, Mitsukuni Sakabe, Kohei Yoshimine, Hinako Kori, Mizuki Muranaka, Hibiki Kiyosue, Motoki Yuasa, Kazunori Tobino

**Affiliations:** 1 Respiratory Medicine, Iizuka Hospital, Iizuka, JPN

**Keywords:** bronchoscopy, diagnostic yield and safety, ebus-tbna, endobronchial ultrasound-transbronchial needle aspiration, pulmonary sarcoidosis, transbronchial lung cryobiopsy

## Abstract

Background: Endobronchial ultrasonography-guided transbronchial needle aspiration (EBUS-TBNA) and transbronchial lung cryobiopsy (TBLC) are widely used to diagnose pulmonary sarcoidosis. However, their combined diagnostic performances and safety profiles have not been comprehensively assessed.

Methods: We retrospectively analyzed 49 consecutive patients who underwent diagnostic bronchoscopy for suspected pulmonary sarcoidosis at a single center between June 2016 and December 2023. Patients were classified into five procedural groups based on the bronchoscopic modality used. The primary outcome was the diagnostic yield, and the secondary outcomes included adverse events and total procedure time.

Results: Histological confirmation was achieved in 87.8% of cases (43/49). The diagnostic yields were 93% for EBUS-TBNA alone, 71% for TBLC alone, and 100% when both were combined. This combination significantly outperformed each technique alone (p=0.003). TBLC did not significantly increase the procedure time or complications. Minor bleeding (grade 1) occurred in 12% of TBLC cases and was effectively managed. The diagnostic yield was positively associated with the number of biopsy samples and specific computed tomography (CT) patterns.

Conclusions: The combination of EBUS-TBNA and TBLC is a safe and highly effective diagnostic approach for pulmonary sarcoidosis, particularly in patients with nodular CT findings. This dual strategy may reduce the need for surgical biopsy and facilitate earlier diagnosis and treatment initiation.

## Introduction

Sarcoidosis is a systemic granulomatous disorder of unknown etiology characterized histologically by non-necrotizing epithelioid cell granulomas [1-3]. Intrathoracic involvement occurs in approximately 90% of patients and is a major determinant of prognosis; the extent of thoracic disease is still commonly described using the chest radiograph-based Scadding (Wurm-Scadding) staging system [2,3]. Given the substantial clinical and imaging overlap with tuberculosis, hypersensitivity pneumonitis, and malignancy, the 2020 American Thoracic Society (ATS) clinical practice guidelines recommend establishing a compatible clinical-radiographic context, obtaining histological confirmation of non-necrotizing granulomas when the tissue is reasonably accessible, and excluding alternative causes before initiating immunosuppressive therapy [1].

Flexible bronchoscopy with forceps transbronchial lung biopsy (TBLB) has long been the standard method for obtaining pulmonary tissue; however, diagnostic yields are highly variable (approximately 40%-90%) owing to small specimen size and crush artifact [4-6], and complications such as pneumothorax (~1.4%) and hemorrhage (~4%) have been reported [7]. Endobronchial ultrasound-guided transbronchial needle aspiration (EBUS-TBNA) has markedly improved the sampling of mediastinal and hilar lymph nodes. Meta-analyses in clinically unselected populations reported a pooled sensitivity of ~84% and specificity of ~100% [6], whereas earlier analyses that pooled studies across settings reported a pooled diagnostic accuracy of ~79% [6,8]. Nevertheless, false-negative results occur, particularly when granulomas are predominantly parenchymal or sparsely distributed; therefore, additional pulmonary sampling is often required if the pretest probability remains high [1,7].

Transbronchial lung cryobiopsy (TBLC) overcomes several limitations of forceps biopsy by yielding larger, architecturally preserved specimens; in sarcoidosis series, diagnostic yields have been reported in roughly two-thirds to over 90% of suspected cases [5,9,10], while broader interstitial lung disease cohorts show pooled yields of ~80%, with pneumothorax and moderate/severe bleeding reported in ~12% and variably up to ~39% of cases, respectively [11].

Prospective comparative data demonstrate the relative strengths of nodal versus parenchymal sampling; in a same-session prospective study of stage I-II sarcoidosis, EBUS-TBNA confirmed the diagnosis in 94% vs. 37% of forceps TBLB [12]. A randomized controlled trial also showed a higher yield of EBUS-TBNA than conventional (blind) TBNA when sampling the same number of nodes and passes (93% vs. 64%) [13], and the multicenter GRANULOMA randomized trial found endosonographic nodal aspiration superior to conventional bronchoscopy (including TBLB and endobronchial biopsy) for stage I/II disease (80% vs. 53%) [1]. As sarcoidosis frequently involves both mediastinal lymph nodes and lung parenchyma, combining nodal and parenchymal sampling can maximize yield: in one series, EBUS-TBNA plus TBLB achieved 100% diagnostic accuracy [7], and a separate cohort suggested a complementary role for pairing EBUS-guided nodal sampling with TBLC [5].

Although both EBUS-TBNA and TBLC are valuable, the diagnostic performance, safety, and efficiency of a pre-specified dual strategy versus either modality alone have not been established in randomized or multicenter prospective studies. Therefore, this study aimed to assess the diagnostic yield, safety profile, and procedural factors associated with EBUS-TBNA, TBLC, and their combined use in a consecutive cohort of patients with suspected pulmonary sarcoidosis [1,5,9].

This article was previously presented as an oral presentation at the 48^th^ Annual Meeting of the Japan Society for Respiratory Endoscopy.

## Materials and methods

Study design and ethics

We conducted a retrospective observational study involving adults (≥ 20 years of age) who underwent diagnostic bronchoscopy for suspected pulmonary sarcoidosis in a single center between June 2016 and December 2023. This study was conducted in accordance with the principles of the Declaration of Helsinki (revised 2013) and was approved by the institutional ethics committee of Iizuka Hospital, Iizuka, Japan (approval number: 24108), which waived the need for written informed consent for the use of medical record data.

Inclusion and exclusion criteria

We included consecutive patients who underwent diagnostic bronchoscopy with EBUS-TBNA and/or TBLC at our institution during the study period. Only cases with a final diagnosis of sarcoidosis, established according to the ATS 2020 diagnostic criteria [3], were analyzed. The final diagnosis was classified as histopathology-confirmed (non-necrotizing granulomas in an appropriate context) or clinical diagnosis (imaging compatible with sarcoidosis plus consistent clinical features and exclusion of alternative causes; supportive bronchoalveolar lavage fluid (BALF) data and follow-up were considered where available). We excluded patients with a final diagnosis other than sarcoidosis, incomplete data, insufficient sample quality, duplicate procedures from the same diagnostic episode, or age <18 years. Because the initial “suspected sarcoidosis” status and alternative final diagnoses were not reliably captured in this retrospective dataset, patients ultimately diagnosed with conditions other than sarcoidosis were not included. Accordingly, the study estimates diagnostic yield among sarcoidosis cases and does not evaluate specificity or false-positive rates.

Clinical data collection from electronic medical records

The following variables were extracted from electronic medical records: age, sex, body mass index (BMI), Scadding stage on chest radiograph, pulmonary function indices (percent predicted forced vital capacity (FVC), forced expiratory volume in 1 second (FEV₁), and diffusing capacity of the lung for carbon monoxide (DLco)), serum biomarkers (C-reactive protein, Krebs von den Lungen-6 (KL-6), surfactant protein-D, angiotensin-converting enzyme, and soluble interleukin-2 receptor), high-resolution computed tomography (HRCT) findings (presence of granular parenchymal opacities and the short-axis diameter of enlarged lymph nodes), and BALF data, including recovery percentage, lymphocyte fraction, and CD4/CD8 ratio.

Bronchoscopic procedures

EBUS-TBNA was performed using a convex-probe bronchoscope (BF-UC290F, Olympus Medical Systems Corp., Tokyo, Japan) in conjunction with 21- or 22-gauge ViziShot aspiration needles from the same manufacturer. Target lymph nodes were selected from mediastinal or hilar nodes identified on chest CT and deemed technically accessible on EBUS. A uniform short-axis threshold of ≥10 mm on CT was used to guide sampling across stations; station-specific “normal diameter” cut-offs were not applied. Selection also considered accessibility and procedural safety. Detailed EBUS sonographic features were not prospectively captured. Most sampled stations were 7, 4, 10, and 11.

TBLC was conducted using either a 1.9 or 2.4 mm cryoprobe connected to the ERBECRYO® 2 system (ERBE Elektromedizin GmbH, Tübingen, Germany). Under fluoroscopic guidance, the cryoprobe was advanced into the predetermined bronchopulmonary segment and activated for 4 s. The cryoprobe-tissue complex was removed en bloc. To mitigate the bleeding risk, a Fogarty arterial embolectomy catheter (model E080-4F, 4 Fr; Edwards Lifesciences, Irvine, CA, USA) was prophylactically inflated as a bronchial blocker during each TBLC procedure. Whenever feasible, two core specimens were obtained from different pulmonary segments, as acquisition of ≥2 samples has been shown to improve the diagnostic yield without significantly increasing procedural risk [11].

BAL was performed in a subset of patients; 40 of 49 underwent BAL, and nine did not due to technical or clinical considerations. When available, BALF differential cell counts and the CD4/CD8 ratio were recorded. BALF results were interpreted as part of an integrated diagnostic assessment together with clinical features and thoracic imaging, in line with the ATS 2020 diagnostic framework [3].

Pathology

All specimens were fixed in 10% neutral buffered formalin, embedded in paraffin, and stained with hematoxylin and eosin (H&E). Additional staining with Ziehl-Neelsen [13] and Grocott’s methenamine silver [14] was performed when infection was suspected. The presence of well-formed, non-caseating granulomas was considered indicative of sarcoidosis.

Outcomes

The primary outcome was diagnostic yield, defined as the proportion of bronchoscopic procedures that resulted in the histological confirmation of sarcoidosis. Secondary outcomes included: (i) the incidence of procedure-related adverse events, graded according to the British Thoracic Society (BTS) criteria [15] for bleeding and pneumothorax, and (ii) the total duration of the bronchoscopy procedure.

Sample‑size rationale

As this was a retrospective study that analyzed all available cases over a defined study period, no a priori sample size calculation was performed.

Statistical analysis

Continuous variables were summarized as median (interquartile range) and categorical variables as counts (percentages). Normality was assessed using the Shapiro-Wilk test and visual inspection of Q-Q plots; several key variables demonstrated non-normal distributions, and homoscedasticity could not be assured. Given the relatively small sample sizes and these assumption violations, between-group comparisons of continuous variables were performed using the Mann-Whitney U test. Categorical variables were compared using the chi-square (χ²) test (with Yates’ continuity correction for 2×2 tables) or Fisher’s exact test when expected cell counts were <10, given the small sample size and sparse contingency tables in this cohort. All statistical analyses were performed using EZR, a graphical interface for R software (R Foundation for Statistical Computing, Vienna, Austria).

## Results

Patient profile

A total of 49 consecutive patients were included in the analysis (Table 1).

**Table 1:** Baseline demographic, functional, and laboratory characteristics of the study cohort Data are presented as N (%) or median (range). Scadding stage refers to the chest radiograph staging system (0/I/II/III/IV). Unless otherwise indicated, N = 49 for all variables; for specific parameters, the sample size (n) is given in parentheses. BMI: body mass index; FVC: forced vital capacity; FEV₁: forced expiratory volume in 1 second; DLco: diffusing capacity for carbon monoxide; CRP: C-reactive protein; KL-6: Krebs von den Lungen-6; SP-D: surfactant protein-D; ACE: angiotensin-converting enzyme; sIL-2R: soluble interleukin-2 receptor; BALF: bronchoalveolar lavage fluid; CD: cluster of differentiation

Characteristics	Value
Age, years	51 (26-80)
Sex	
Male	24 (49)
Female	25 (51)
BMI, kg/m²	23.1 (17.1-39.3)
Scadding stage	
0	11 (22)
I	18 (37)
II	14 (29)
III	2 (4)
IV	4 (8)
FVC, % predicted (n = 32)	97.1 (38.8-121.5)
FEV₁, % predicted (n = 32)	82.1 (55.5-100)
DLco, % predicted (n = 16)	91.2 (50-118)
CRP, mg/dL	0.13 (0.02-6.10)
KL-6, U/mL (n = 26)	321 (129-4622)
SP-D, ng/mL (n = 20)	64 (15-920)
ACE, IU/L	23.1 (12.1-232)
sIL-2R, U/mL	795 (189-3530)
BALF lymphocyte ratio, % (n = 40)	30 (1-76)
BALF CD4/CD8 ratio (n = 40)	3.9 (0.4-28.0)
Diagnosis type	
Histological diagnosis	43 (88)
Clinical diagnosis	6 (12)

The median age was 51 years (range: 26-80 years), and the sex distribution was nearly equal (24 men and 25 women). Pulmonary function was generally preserved, with a median percent predicted FVC of 97.1% (range: 38.8%-121.55%) and a median DLco of 91.2% (range: 50%-118%). Based on chest radiographic staging, 37% of patients were classified as Scadding stage I and 29% as stage II. Among patients who underwent BAL (n = 40), the BALF lymphocyte fraction had a median of 30% (range: 1-76%), and the CD4/CD8 ratio had a median of 3.9 (range: 0.4-28). Given missingness and sample size, these data are presented descriptively without formal hypothesis testing. BALF variables are also summarized in Table 1.

Sampling strategies and overall yield

**Figure 1:**
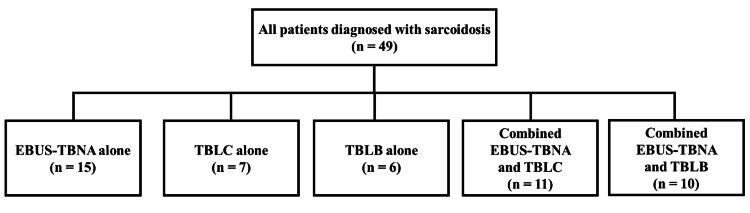
Distribution of bronchoscopic tissue-sampling modalities in patients with pulmonary sarcoidosis Flow diagram of 49 study patients with histologically confirmed sarcoidosis, categorized by the bronchoscopic diagnostic technique employed: endobronchial ultrasound-guided transbronchial needle aspiration (EBUS-TBNA) alone (n = 15), transbronchial lung cryobiopsy (TBLC) alone (n = 7), forceps transbronchial lung biopsy (TBLB) alone (n = 6), combined EBUS-TBNA + TBLC (n = 11), and combined EBUS-TBNA + TBLB (n = 10).

Each patient underwent a single bronchoscopic evaluation and was assigned to one of the following five procedural groups (Figure 1): EBUS-TBNA alone (n = 15), TBLC alone (n = 7), conventional TBLB alone (n = 6), combined EBUS-TBNA + TBLC (n = 11), or EBUS-TBNA + TBLB (n = 10).

Histological confirmation of sarcoidosis was achieved in 43 of 49 patients, yielding an overall diagnostic rate of 88%. Table 2 presents the 2×2 contingency data and corresponding sensitivity estimates for each single and combined sampling modality.

**Table 2:** Diagnostic accuracy of each bronchoscopic sampling modality for pulmonary sarcoidosis—cross-tabulation and sensitivity estimates. All 49 participants satisfied the reference standard (histology positive for non-caseating granulomas); no reference-negative cases occurred, so specificity and predictive values cannot be calculated. EBUS-TBNA: endobronchial ultrasound-guided transbronchial needle aspiration; TBLC: transbronchial lung cryobiopsy; TBLB: forceps transbronchial lung biopsy; Ref: reference standard

Sampling modality	N	True-positive (Ref +, Test +)	False-negative (Ref +, Test –)	Sensitivity %	True-negative/False-positive*
EBUS-TBNA	36	26	10	72%	0 / 0
TBLC	18	16	2	89%	0 / 0
TBLB	16	6	10	38%	0 / 0
EBUS-TBNA + TBLC	11	11	0	100%	0 / 0
EBUS-TBNA + TBLB	10	9	1	90%	0 / 0

In stand-alone tests, EBUS-TBNA detected non-caseating granulomas in 14 of 15 patients (93%), TBLC in five of seven (71%), and conventional TBLB in two of six (33%). Concurrent EBUS-TBNA plus TBLC diagnosed all 11 patients (100%), a performance significantly superior to either index test alone (χ² = 11.6, p=0.003). EBUS-TBNA paired with conventional TBLB also achieved a high yield (9/10, 90%), although its sensitivity was lower than that of the TBLC-enhanced strategy.

Effect of biopsy number

**Figure 2:**
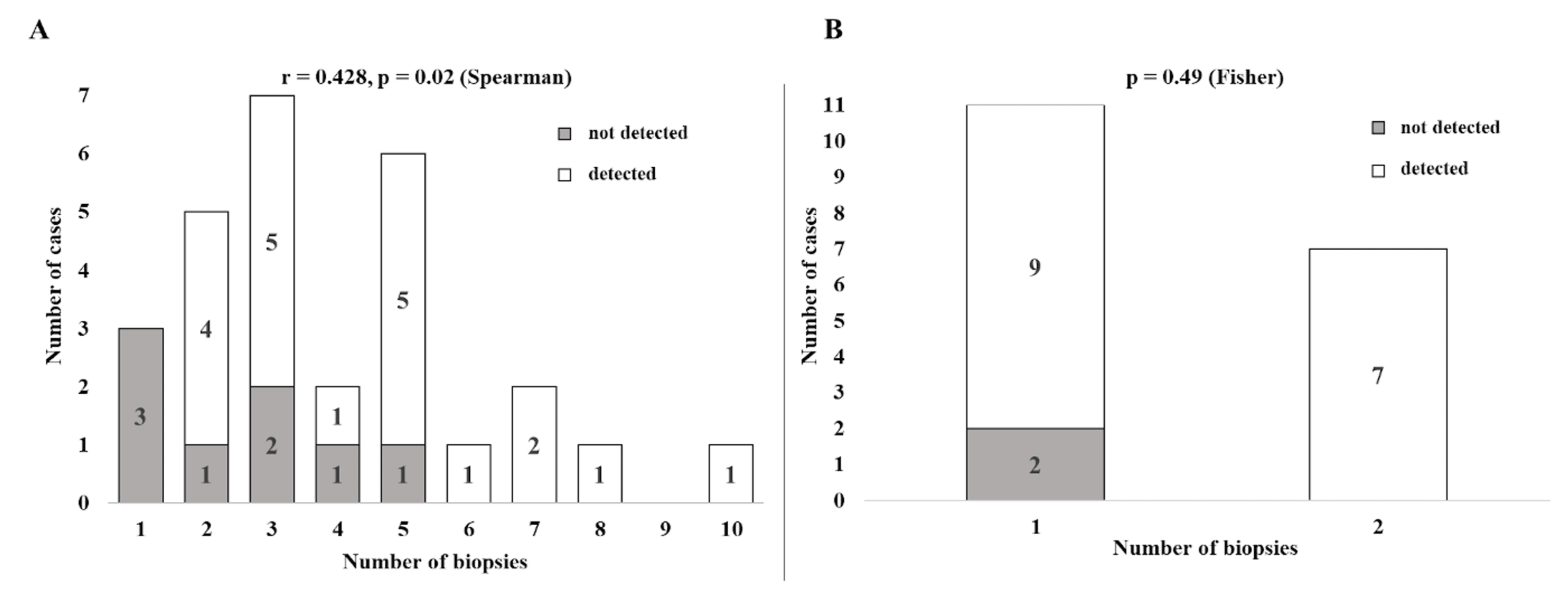
Diagnostic yield of EBUS-TBNA and TBLC stratified by the number of biopsy specimens obtained A (EBUS-TBNA): Histogram showing the number of cases in which non-caseating granulomas were detected (white bars) or not detected (gray bars) according to the number of lymph node aspirations performed during EBUS-TBNA. Table 3 shows the detection rates (%) for each biopsy count. A positive correlation was observed between the number of aspirations and the diagnostic yield (Spearman’s r=0.428, p=0.02). B (TBLC): Histogram depicting the diagnostic yield of TBLC when one or two cryobiopsy samples are obtained. Detection (white bars) and non-detection (gray bars) of granulomas are indicated, with the corresponding detection rates shown in Table 3. There was no significant difference in yield between one and two cryobiopsies (Fisher’s exact test, p=0.49). EBUS-TBNA: endobronchial ultrasound-guided transbronchial needle aspiration; TBLC: transbronchial lung cryobiopsy

Increasing the number of tissue samples was associated with an improved diagnostic yield. In the case of EBUS-TBNA, sensitivity increased from 60% with a single needle pass to ≥80% after two passes, and reached 100% when five or more passes were performed. The correlation between the number of passes and diagnostic success was moderate but statistically significant (r=0.428, p=0.02; Spearman’s rank correlation test; Figure 2A and Table 3).

**Table 3:** Association between the number of biopsy samples and diagnostic yield EBUS-TBNA: endobronchial ultrasound-guided transbronchial needle aspiration; TBLC: transbronchial lung cryobiopsy

Variable	EBUS-TBNA										TBLC	
Number of biopsies	1	2	3	4	5	6	7	8	9	10	1	2
Detection rate, %	0	80	71	50	83	100	100	100	-	100	82	100

For TBLC, the diagnostic yield was 82% for one core sample and 100% for two cores. However, this difference was not statistically significant (p=0.49, Fisher’s exact test; Figure 2B and Table 3).

Predictors of EBUS‑TBNA success

**Figure 3:**
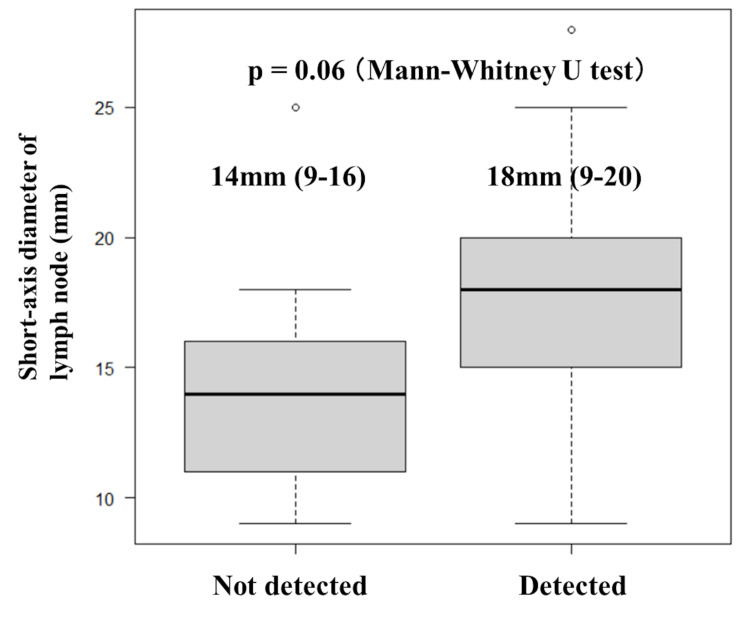
Short-axis diameter of sampled lymph nodes stratified by EBUS-TBNA diagnostic outcome Box-and-whisker plots show the short-axis diameters (mm) of the mediastinal and hilar lymph nodes sampled using endobronchial ultrasound-guided transbronchial needle aspiration (EBUS-TBNA). The median (range) values are annotated above each box (detected: 18 mm (9–20 mm) vs. not detected: 14 mm (9–16 mm)). The Mann-Whitney U test revealed no statistically significant differences between the groups (p=0.06).

Lymph node size on CT appeared to influence the diagnostic yield. Nodes that yielded granulomas had a larger median short-axis diameter of 18 mm (range: 9-20 mm) than non-diagnostic nodes, which had a median diameter of 14 mm (range: 9-16 mm). This difference approached statistical significance (p=0.06; Mann-Whitney U test; Figure 3).

CT pattern and TBLC yield

**Figure 4:**
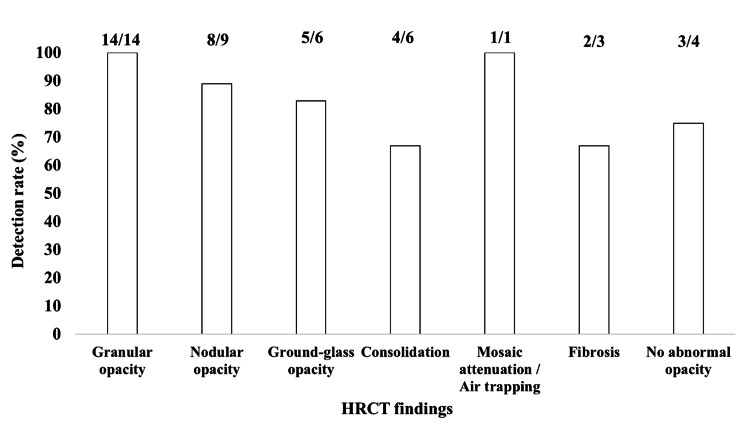
Diagnostic yield of transbronchial lung cryobiopsy stratified by a predominant HRCT pattern Bar chart showing the proportion of biopsy specimens containing non-necrotizing granulomas for each high-resolution CT (HRCT) feature in patients evaluated for pulmonary sarcoidosis. The y-axis shows the detection rate (%) calculated for granulomas/biopsy samples, whereas the numerical fractions above each bar provide the absolute values for that ratio. CT patterns were classified as granular opacity, nodular opacity, ground-glass opacity, consolidation, mosaic attenuation/air trapping, fibrosis, or no abnormal opacity. Granular opacities (14/14) and mosaic attenuation/air trapping (1/1) yielded 100% detection, whereas consolidation (4/6) and fibrosis (2/3) showed lower yields of 66.7%. These results suggest that targeting lesions with granular or nodular opacities may maximize histological diagnostic yield during cryobiopsy.

TBLC procedures targeting granular or nodular parenchymal opacities in CT-diagnosed sarcoidosis in all 14 evaluable cases (100%). In contrast, cryobiopsies directed at apparently normal lung parenchyma still yielded a diagnostic result in three out of four cases (75%). Ground-glass, reticular, or consolidative patterns demonstrated intermediate diagnostic yields ranging from 67% to 89% (Figure 4).

Among 25 traceable cryobiopsy specimens, granulomas were identified in four of five upper lobe samples (80%), the single middle lobe sample (100%), and all nine lower lobe samples (100%), suggesting no statistically significant lobar difference (p=0.27).

Procedure duration and safety

Table 4 shows the detailed breakdown of procedure durations and complication status by procedure group.

**Table 4:** Procedure duration and safety Data are presented as median (range). No procedure-related complications (e.g., pneumothorax, hemorrhage grade ≥2) were observed in any group. EBUS-TBNA: endobronchial ultrasound–guided transbronchial needle aspiration; TBLB: transbronchial lung biopsy; TBLC: transbronchial lung cryobiopsy

Procedure group	Duration of procedure, min (median (range))	Number of patients (n)	Complications observed
EBUS-TBNA alone	47 (18-60)	15	None
TBLC alone	49 (25-58)	7	None
TBLB alone	29 (16-34)	6	None
Combined EBUS-TBNA and TBLC	49 (34-70)	11	None
Combined EBUS-TBNA and TBLB	63 (26-93)	10	None

The median total bronchoscopy duration did not significantly differ among the procedural groups: 46.5 min for EBUS-TBNA alone, 49.1 min for TBLC alone, and 49.2 min for the combined EBUS-TBNA + TBLC procedure (p=0.831). No pneumothorax, infection, or hemodynamic instability was observed. Minor bleeding (grade 1) occurred after three TBLC procedures (12%) and was promptly controlled with a prophylactically placed Fogarty balloon. No grade ≥2 bleeding events were recorded, and EBUS-TBNA was not associated with any clinically significant hemorrhage.

## Discussion

Our dual-compartment strategy, sampling mediastinal/hilar lymph nodes with EBUS-TBNA and the parenchyma with TBLC in the same session, yielded histological confirmation in all patients. This mirrors the 100% sensitivity originally reported for the TBLC + EBUS-TBNA approach [6], underscoring the complementary value of combined nodal and parenchymal sampling. Meta-analyses found the pooled sensitivity of EBUS-TBNA for sarcoidosis between 79% and 84% in unselected cohorts [6,8]. Our findings confirm this and add that larger short-axis lymph node diameters independently predict a positive result, which is consistent with prior determinant analyses [16].

Cryobiopsy yielded diagnostic tissue in 71% of stand-alone cases in our series, comparable to the 67%-93% range reported in single-center sarcoidosis cohorts [6,9,10]. Published systematic reviews of interstitial lung disease populations show pooled yields of around 80% [11].

In our cohort, minor bleeding occurred in 12% of TBLCs, and no pneumothorax was observed, aligning with contemporary systematic reviews that reported pneumothorax and moderate-to-severe bleeding in approximately 12% and 39% of procedures, respectively [11]. Importantly, procedural proficiency and complication rates improve with experience. A learning-curve study demonstrated that diagnostic yield and sample size improve with experience, with concurrent declines in pneumothorax incidence [10].

Approximately 10%-40% of patients with sarcoidosis progress to a fibrotic phenotype associated with reduced quality of life and survival [3,17]; early, minimally invasive confirmation, therefore, remains critical. EBUS-TBNA is recognized as being more cost-effective than mediastinoscopy for intrathoracic tissue acquisition [1]. The present data suggest that adding TBLC rarely prolongs bronchoscopy, yet obviates surgical lung biopsy in most stage I/II cases, a finding likely to improve the overall cost-utility, although formal modeling is warranted.

Newer adjuncts, including EBUS-guided intranodal forceps biopsy and cryo-EBUS mediastinal cryobiopsy, have shown additive diagnostic benefits in benign adenopathy and lymphoma and may further augment sensitivity when conventional aspirations are non-diagnostic [18,19]. Prospective head-to-head trials comparing TBLC-enhanced protocols with these evolving techniques are required to define the optimal sequencing.

In sarcoidosis, BALF often shows lymphocytosis; however, compared with hypersensitivity pneumonitis, the CD4/CD8 ratio tends to be higher, and characteristic imaging findings such as perilymphatic nodules or bilateral hilar lymphadenopathy help differentiate sarcoidosis from hypersensitivity pneumonitis and other interstitial lung diseases. Although non-caseating granulomas are not pathognomonic for sarcoidosis and may also appear as sarcoid reactions in other diseases, the combination of these radiological and immunological findings made the diagnosis of sarcoidosis more plausible in this study.

This study has several limitations. First, it was conducted at a single center with a relatively small sample size, which may limit generalizability. Second, the retrospective design may have introduced selection bias and unmeasured confounding. Third, diagnostic procedures and clinical decisions were not fully standardized and reflected institutional practice, which may reduce reproducibility across centers. Fourth, by design, we included only patients with a final diagnosis of sarcoidosis; this restriction may introduce spectrum bias and exclude patients who were initially suspected of sarcoidosis but ultimately had other diseases. As a result, specificity and false-positive rates could not be assessed. We note that granulomas obtained by EBUS-TBNA can represent sarcoid-like reactions associated with malignancy, and TBLC can show granulomas in hypersensitivity pneumonitis. Finally, long-term outcomes were not fully assessed. Future prospective multicenter studies that enroll all clinically/CT-suspected cases and adjudicate final diagnoses are needed to validate and extend these findings.

## Conclusions

Our retrospective single-center experience suggests that the combined use of EBUS-TBNA and TBLC is a safe and effective bronchoscopic strategy to maximize diagnostic yield in suspected pulmonary sarcoidosis. When radiologic findings and procedural access permit, we recommend employing ≥3 nodal passes and ≥2 cryobiopsies as a pragmatic default. Prospective multicenter trials are warranted to refine optimal biopsy parameters, establish CT-based targeting algorithms, and assess cost-effectiveness across diverse healthcare settings.

## References

[1] Crouser ED, Maier LA, Wilson KC (2020). Diagnosis and detection of sarcoidosis. An official American Thoracic Society clinical practice guideline. Am J Respir Crit Care Med.

[2] Iannuzzi MC, Rybicki BA, Teirstein AS (2007). Sarcoidosis. N Engl J Med.

[3] Belperio JA, Fishbein MC, Abtin F, Channick J, Balasubramanian SA, Lynch Iii JP (2024). Pulmonary sarcoidosis: a comprehensive review: past to present. J Autoimmun.

[4] Häntschel M, Eberhardt R, Petermann C (2021). Diagnostic yield of transbronchial lung cryobiopsy compared to transbronchial forceps biopsy in patients with sarcoidosis in a prospective, randomized, multicentre cross-over trial. J Clin Med.

[5] Aragaki-Nakahodo AA, Baughman RP, Shipley RT, Benzaquen S (2017). The complimentary role of transbronchial lung cryobiopsy and endobronchial ultrasound fine needle aspiration in the diagnosis of sarcoidosis. Respir Med.

[6] Trisolini R, Lazzari Agli L, Tinelli C, De Silvestri A, Scotti V, Patelli M (2015). Endobronchial ultrasound-guided transbronchial needle aspiration for diagnosis of sarcoidosis in clinically unselected study populations. Respirology.

[7] Plit M, Pearson R, Havryk A, Da Costa J, Chang C, Glanville AR (2012). Diagnostic utility of endobronchial ultrasound-guided transbronchial needle aspiration compared with transbronchial and endobronchial biopsy for suspected sarcoidosis. Intern Med J.

[8] Navasakulpong A, Auger M, Gonzalez AV (2016). Yield of EBUS-TBNA for the diagnosis of sarcoidosis: impact of operator and cytopathologist experience. BMJ Open Respir Res.

[9] Jacob M, Bastos HN, Mota PC (2019). Diagnostic yield and safety of transbronchial cryobiopsy in sarcoidosis. ERJ Open Res.

[10] Davidsen JR, Skov IR, Louw IG, Laursen CB (2021). Implementation of transbronchial lung cryobiopsy in a tertiary referral center for interstitial lung diseases: a cohort study on diagnostic yield, complications, and learning curves. BMC Pulm Med.

[11] Johannson KA, Marcoux VS, Ronksley PE, Ryerson CJ (2016). Diagnostic yield and complications of transbronchial lung cryobiopsy for interstitial lung disease. A systematic review and metaanalysis. Ann Am Thorac Soc.

[12] Oki M, Saka H, Kitagawa C, Kogure Y, Murata N, Ichihara S, Moritani S (2012). Prospective study of endobronchial ultrasound-guided transbronchial needle aspiration of lymph nodes versus transbronchial lung biopsy of lung tissue for diagnosis of sarcoidosis. J Thorac Cardiovasc Surg.

[13] (2025). SOP for Ziehl-Nielson staining. https://extranet.who.int/lqsi/content/tb-sop-ziehl-nielson-staining.

[14] Wang S, Lai J, Wu R (2022). Grocott methenamine silver staining is the optimal approach to histological diagnosis of pulmonary cryptococcosis. Front Microbiol.

[15] Du Rand IA, Blaikley J, Booton R (2013). British Thoracic Society guideline for diagnostic flexible bronchoscopy in adults: accredited by NICE. Thorax.

[16] Li K, Jiang S (2014). A randomized controlled study of conventional TBNA versus EBUS-TBNA for diagnosis of suspected stage I and II sarcoidosis. Sarcoidosis Vasc Diffuse Lung Dis.

[17] Bandyopadhyay D, Mirsaeidi MS (2023). Sarcoidosis-associated pulmonary fibrosis: joining the dots. Eur Respir Rev.

[18] Zhang Z, Li S, Bao Y (2024). Endobronchial ultrasound-guided transbronchial mediastinal cryobiopsy versus endobronchial ultrasound-guided transbronchial needle aspiration for mediastinal disorders: a meta-analysis. Respiration.

[19] Mangold MS, Franzen DP, Hetzel J (2024). Ultrasound-guided transbronchial cryobiopsy of mediastinal and hilar lesions: a multicenter pragmatic cohort study with real-world evidence. BMJ Open Respir Res.

